# Quantifying the spatial risk of Avian Influenza introduction into British poultry by wild birds

**DOI:** 10.1038/s41598-019-56165-9

**Published:** 2019-12-27

**Authors:** Andrew Hill, Simon Gillings, Alexander Berriman, Adam Brouwer, Andrew C. Breed, Lucy Snow, Adam Ashton, Charles Byrne, Richard M. Irvine

**Affiliations:** 1 Animal and Plant Health Agency, New Haw, KT15 3NB United Kingdom; 2 British Trust for Ornithology, Thetford, IP24 2PU United Kingdom; 3 Present Address: University of Surrey, Guildford, UK; 4 Present Address: School of Veterinary Science, University of Queensland, Brisbane, Australia; 5 Epidemiology and One Health Section, Department of Agriculture, Canberra, Australia

**Keywords:** Zoology, Applied mathematics

## Abstract

The transmission of pathogens across the interface between wildlife and livestock presents a challenge to the development of effective surveillance and control measures. Wild birds, especially waterbirds such as the Anseriformes and Charadriiformes are considered to be the natural hosts of Avian Influenza (AI), and are presumed to pose one of the most likely vectors for incursion of AI into European poultry flocks. We have developed a generic quantitative risk map, derived from the classical epidemiological risk equation, to describe the relative, spatial risk of disease incursion into poultry flocks via wild birds. We then assessed the risk for AI incursion into British flocks. The risk map suggests that the majority of AI incursion risk is highly clustered within certain areas of Britain, including in the east, the south west and the coastal north-west of England. The clustering of high risk areas concentrates total risk in a relatively small land area; the top 33% of cells contribute over 80% of total incursion risk. This suggests that targeted risk-based sampling in a relatively small geographical area could be a much more effective and cost-efficient approach than representative sampling. The generic nature of the risk map method, allows rapid updating and application to other diseases transmissible between wild birds and poultry.

## Introduction

Avian Influenza (AI) viruses are of great concern as livestock pathogens in terms of both animal welfare and economic impacts, but also as potential zoonoses and progenitors of future pandemic disease. These viruses can move between wildlife, poultry and human populations. The subtypes of most concern for incursion into poultry flocks are H5 and H7, where Highly Pathogenic AI (HPAI) pathotypes can mutate from Low Pathogenicity AI (LPAI) viruses, leading to systemic infection of susceptible species of poultry and subsequently severe morbidity and mortality^1^. Therefore, both HPAI and LPAI subtypes of H5 and H7 are notifiable diseases to the World Organisation for Animal Health (Office International des Epizooties, OIE) and European Commission (EC), and once detected are subject to control measures by both national and international bodies^2,3^. Wild waterbirds such as Anseriformes (ducks, swans and geese) and Charadriiformes (shorebirds, gulls and terns) are considered to be the natural reservoir of influenza A viruses, and their interaction with poultry is a risk factor for the introduction of AI into poultry^4,5^. Following the emergence and global spread of the so-called Eurasian lineage H5N1 HPAI viruses since 1996, the European Commission have required Member States (MSs) to undertake statutory surveillance programmes for the detection and “early warning” of H5N1 HPAI in wild birds to mitigate the risk of infection of poultry^6^.

In April 2006, H5N1 HPAI was isolated from a Whooper swan (Cygnus cygnus) that had been found dead on the east coast of Scotland, marking the first detection of the so-called Eurasian lineage H5N1 HPAI virus in Great Britain (GB)^7^. This led to the development of a risk map to assess the risk of H5N1 HPAI introduction to British poultry flocks^8^. The map from this model was recently updated using more recent poultry demographic data and wild bird abundance data^9^. The model output was a summed risk score across all poultry and wild bird species for each 10 × 10 km grid square^10^. The resulting risk map was used by the United Kingdom (UK) Government to enable prioritisation of AI surveillance activities, including the collection and testing of dead wild birds for H5N1 HPAI. The previous model was developed quickly as a risk prioritisation tool based on the knowledge and available data in 2005–2006, which led to a heavy reliance on (i) expert opinion and (ii) the integration of several wild bird datasets that had non-consistent methodologies. Due to research into the epidemiology of LPAI and HPAI since 2006, and the availability of new data sources, we have been able to develop a quantitative approach for assessing the relative spatial risk of AI viruses into poultry. We have therefore refined and extended the previously developed model by (i) replacing expert opinion with data from observations, (ii) replacing the arbitrary, expert-opinion-derived scoring framework with a quantitative epidemiological framework, and (iii) broadened the scope to include all HPAI and LPAI strains. The resulting risk map method is a generic method applicable to all countries, and all pathogens with similar transmission characteristics to AI (e.g. both airborne and fomite transmission). We have simply parameterised the risk map for the risk of AI introduction into the UK poultry flock through wild birds.

## Methods

### Risk question and scope

The specific risk question was: How does the spatial risk of HPAI and LPAI incursion in poultry via wild birds vary across Great Britain (GB)? It was not possible to estimate absolute risk due to a lack of knowledge regarding the amount of contact between wild birds and poultry; hence we measured relative spatial risk on a 10 × 10 km Ordnance Survey (OS) grid^10^. This was appropriate to the spatial resolution of the poultry and wild bird datasets used, especially with respect to the ranging behaviour of wild birds. Poultry flocks were defined as indoor and outdoor and species (chicken, turkey, duck, partridges, pheasants and goose holdings) across GB as recorded in the official British poultry demographic database.

### Theoretical model framework

We have chosen a generic method based on the classical epidemiological risk equation^11^ of the form $R(t)=1-{e}^{-{I}_{D}(t)}$, where the risk *R*(*t*) is defined as the proportion of the at-risk population affected in time period *t* ∈ [*t*_0_, *t*′] given incidence rate *I*_*D*_(*t*). We then make the model specific for AI and poultry by applying relevant parameter estimates. This methodology has the advantage of (i) being based on the epidemiological definition of risk, (ii) being quick to update (new parameter estimates are inputted as they become available), and (iii) widely applied to other pathogens transmitted between wild birds and poultry.

Thus, the risk of AI introduction to a poultry farm of type *j*, *R*(*j*, *t*), can be defined as the proportion of farms within a grid cell that becomes infected during some time period *t*, that is

1 $$R(j,t)=1-\mathop{\sum }\limits_{{t}_{0}}^{t^{\prime} }\,{e}^{-{I}_{D}(j,t)},$$

where the incidence rate is the number of newly infected farms during time *t*. The number of new infections during *t* can be evaluated by the classical disease transmission equation^12^,

2 $${I}_{D}(j,t)=\beta (j)S(j,t)I(t).$$

where *β*(*j*) is the transmission parameter, and *S*(*j*, *t*) is the number of susceptible poultry flocks of type *j* and *I*(*t*) is the number of infected wild birds within the cell at time *t*. We assume that each 10 × 10 km grid square (cell) is essentially a closed ecosystem (that is, there is no contact between different grid cells), and that a poultry holding is the epidemiological unit of interest. The number of infected wild birds is broken down further, so that *I*(*t*) = *Ap*. The parameter *A* is the relative abundance of wild birds in a cell and *p* is the prevalence of AI infection in wild birds (we assume a constant prevalence over all cells and wild bird species). Therefore,

3 $$R(t)=1-\sum _{j}\,\mathop{\sum }\limits_{{t}_{0}}^{t^{\prime} }\,{e}^{-\beta (j)S(j,t)I(t)},$$

The most uncertain parameter is *β*(*j*), the rate of infection per susceptible domestic flock of type *j*. The absolute value of *β*(*j*) is difficult to reliably determine from surveillance data as there is limited knowledge of the interaction between infected wild birds (and other potential sources of infection) and poultry flocks. However, we may more reliably assess the relative risks between production types (that is, the difference between *β*(*j*) for each poultry type). As such, the value of *R*(*j*) for each grid square is considered a relative risk estimator, not an accurate representative of absolute risk. For the purposes of risk-based surveillance, a relative risk estimator is sufficient as this can be used to weight the sampling of poultry flocks by spatial and production type considerations.

### Parameter estimation

#### Poultry flock density

**Figure 1:**
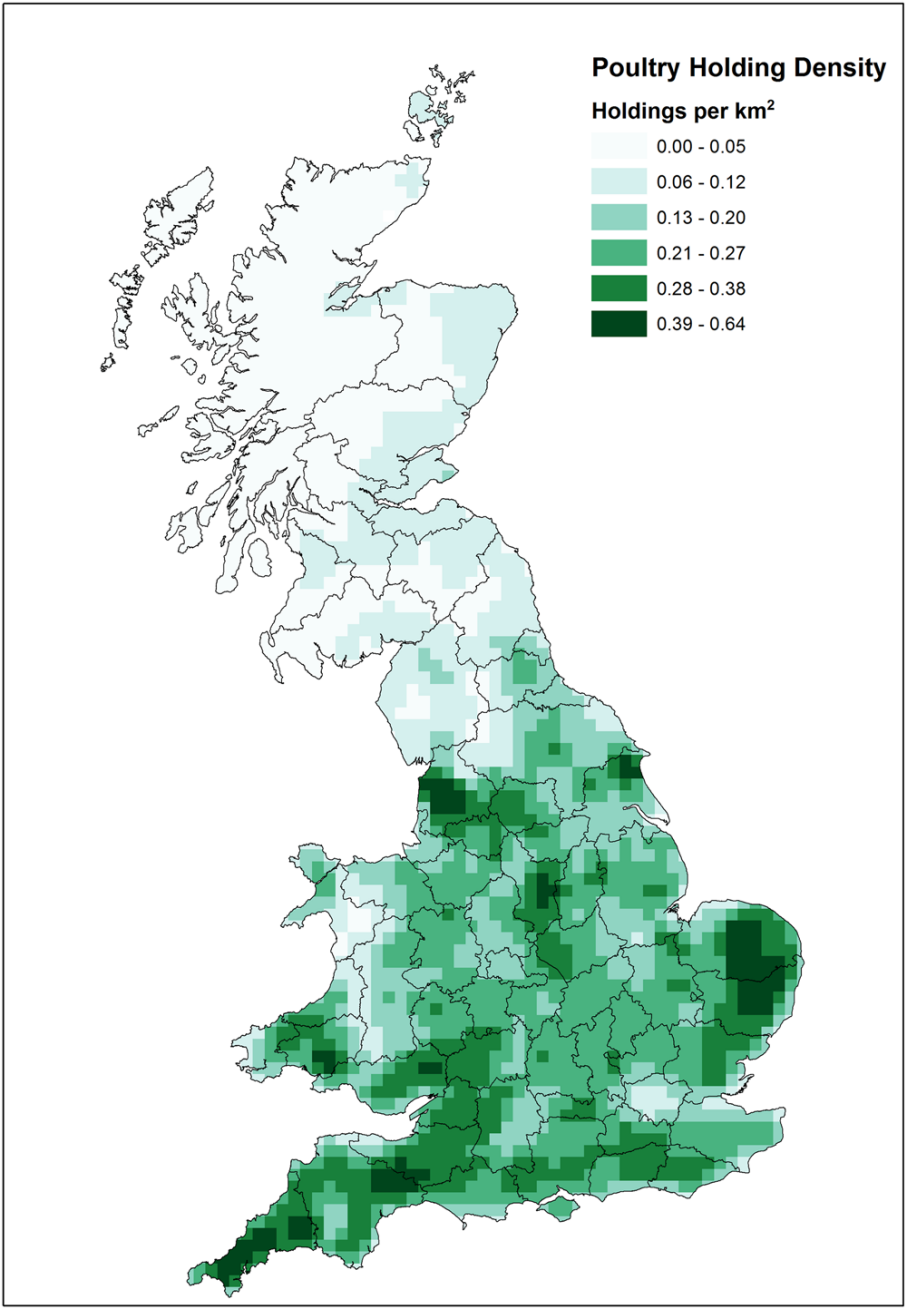
UK poultry holding density as of April 2016^13^. Map was created in ArcGIS Desktop 10.2 (ESRI,Redlands, CA).

The number and density of susceptible poultry flocks was assessed by OS 10 × 10 km grid squares using poultry demographic data in GB collected through an official animal health management database (SAM) maintained by the Animal and Plant Health Agency (a summarised density map of poultry holdings is shown in Fig. 1, but in the model we have produced a density map for each production type *j*). This database includes information on location and other epidemiological and husbandry system data (as at April 2016)^13^. To include individual holdings in the analysis, we used the following information: poultry species; usual stock numbers; and an indicator of whether production was indoors or outdoors. Many farms indicate both indoor and outdoor production; we assumed that any indication of outdoor production increased the risk of exposure to wild birds, and so all farms with mixed husbandry systems were categorised as having outdoor production.

#### Wild bird density

**Figure 2:**
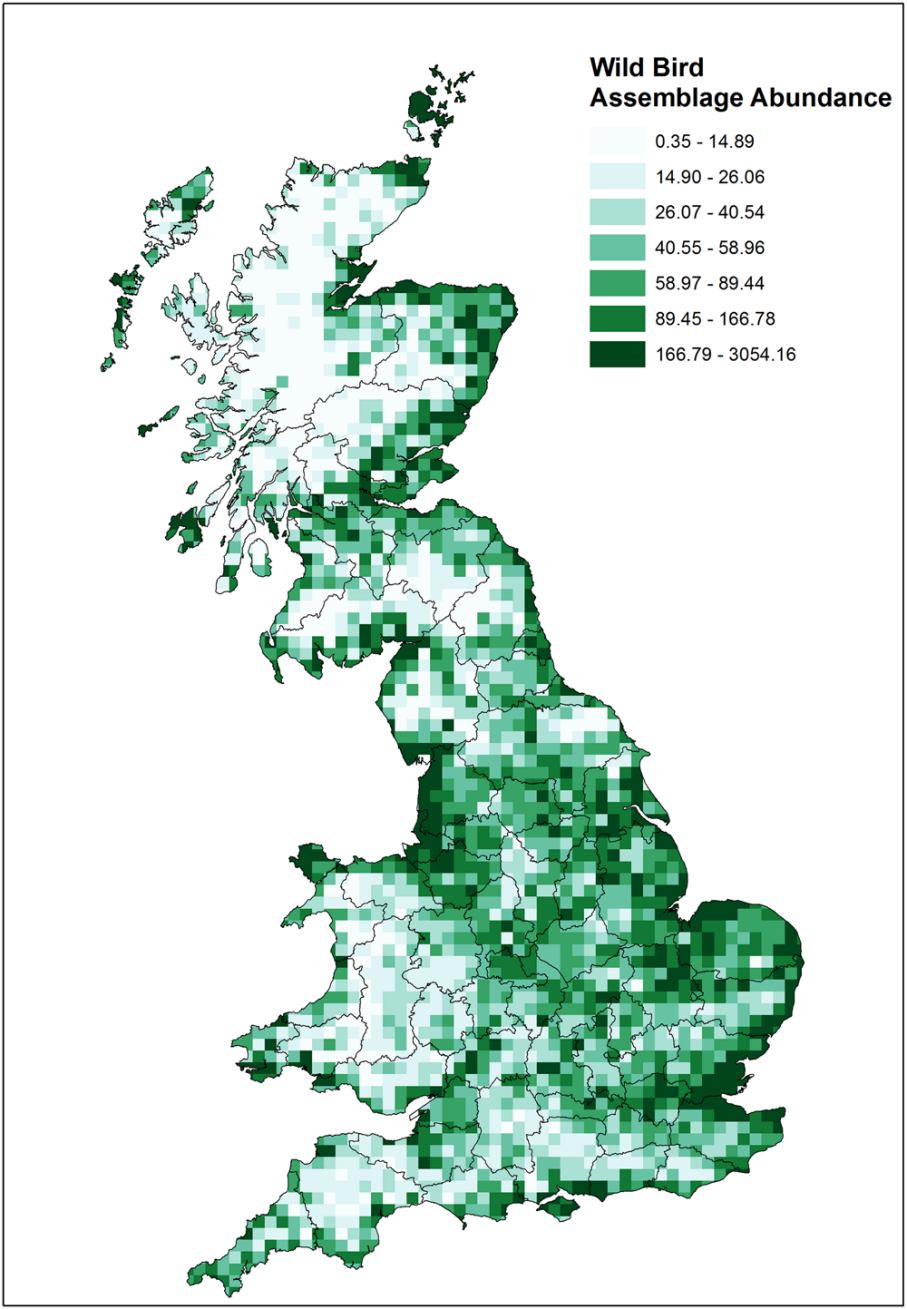
Wild bird assemblage abundance in GB, derived from 109 species considered most relevant for the transmission of AI to poultry flocks (as described in Supplementary Information. Map was created in ArcGIS Desktop 10.2 (ESRI,Redlands, CA).

The assessment of wild bird abundance has changed markedly from the previous study by Snow *et al*.^8^ (see Fig. 2), due to both a change in the focus of the assessment (species that could be infected with H5N1 HPAI to species that could be infected with any LPAI or HPAI) and improvements in the availability of robust and representative data of the location and abundance of wild birds in GB.

The starting point for the selection of wild bird species relevant for the introduction of either HPAI or LPAI into British poultry flocks was the assessment of population sizes for regularly occurring breeding and wintering species in Great Britain and the United Kingdom produced by Musgrove *et al*.^14^. Of the 290 species and populations identified, we extracted information for waterbirds, their predators and scavengers that could conceivably consume waterbird remains. We define waterbirds as the Anseriformes (ducks, geese and swans) and the Charadriiformes (waders, gulls, terns and auks). From these groups we excluded the Sternidae (terns) and Alcidae (auks) because members of these families are exclusively or largely marine and unlikely to contribute to terrestrial spread of AI. Finally we considered birds of prey (order Accipitriformes) and the crows (family Corvidae) as potential predators and scavengers. Note that the gulls (family Laridae) qualify both as members of the Charadriformes and as potential scavengers. In total we considered 109 species - see the Supplementary Information for the individual species assessments.

For each species we extracted current estimates of breeding and wintering population size from Musgrove *et al*.^14^. Most breeding population estimates were published in units of the number of pairs or territories present, which we convert to the number of individuals (assuming two birds per territory) to compare with estimates of winter population size. Where the published population estimate was a range (minima and maxima) we used the midpoint. Species not present in a particular season are highlighted (coded NP). Some species (coded NE) were only assigned a breeding population estimate in Musgrove *et al*.^14^, typically because they are resident breeders and monitoring is based on breeding-season surveys. For these species the winter population size will be at least as large as the breeding population, subject to productivity, post-fledging survival and natal dispersal.

For the 109 species selected, the following criteria were used to further sift species for inclusion in wild bird abundance calculations:

- Waterbird abundance - a minimum of >1000 individuals in GB for Anseriformes and Charadriformes;
- Predator or scavenger abundance - a minimum of >100 individuals in GB;
- Diet:

- Predatory species were classified according to whether waterbirds comprised a significant part of their diet;
- Whether carrion formed a significant part of the diet;

- Habitat use - do significant numbers occupy terrestrial and freshwater habitats during at least part of the year?

The rationale for these criteria was to (i) exclude scarce species and vagrants that are highly localised or essentially random in their occurrence pattern; (ii) exclude predators and scavengers that are so scarce that the likelihood of them encountering infected waterbirds is negligible; (iii) only consider predators and scavengers that could potentially ingest AI infected birds; and (iv) exclude species with largely or entirely coastal, intertidal or marine distributions which are unlikely to occur in terrestrial systems and come into contact with poultry. This is necessary because at 10-km resolution, some species appear to occupy squares dominated by agriculture when in reality they actually occur in the narrow coastal fringe. Where criteria are subjective - for example carrion feeding - we relied on our subject matter expertise.

These criteria were successful for the majority of species but we made two minor adjustments. First, the White-tailed Eagle (Haliaeetus albicilla) breeding population did not meet the 100 individuals threshold but, as a late-maturing species, a large number of sub-adult and non-breeding birds also occur which will likely take the actual population size of potential scavengers over the 100 bird threshold. Second, Whimbrel (Numenius phaeopus) is a common passage migrant, with birds occurring in terrestrial landscapes during spring on route from wintering grounds in Africa to breeding grounds in Iceland and Fennoscandia. No figures for passage population size exist, but with over 600,000 individuals breeding in Iceland, and over 190,000 in Fennoscandia^15^, the potential for significant numbers passing through Britain is considerable.

We did not use either season or origin as criteria. We did not restrict the selection to passage and wintering species owing to the interest in assessing population of waterbirds at other times of the year which may act as a reservoir for low pathogenicity AI. We did not restrict the selection to species originating from Asia because species from other breeding areas (e.g. Canadian arctic, Iceland, Greenland) winter in the UK in large numbers, whereupon they regularly move among different regions. They have the potential to act as carriers of AI within the UK.

#### Probability of AI introduction into a poultry flock

The probability of AI introduction into poultry flocks from wild birds (or other sources) is hard to determine accurately, although estimates can be inferred from active and scanning surveillance of poultry. The risk-based sampling scheme of the British poultry survey^16^ makes it difficult to assess relative risks between production types, especially for those rarely sampled such as indoor chicken breeding flocks. Hence, of particular interest is the EC-mandated AI surveillance programme in the Netherlands^17,18^, which requires that every poultry flock is sampled at least once per year. This means that these data should be highly robust and representative.

The Gonzales study^17,18^ estimated the relative risks (RRs) between Dutch flocks of different species and production types, compared to a baseline, low rate of introduction into indoor egg-laying chicken flocks per month, P(indoor chicken) (no route of introduction is assumed). That is, *P*(*x*) = P(indoor chicken) × *RR*(*x*), where *x* is the Dutch production system. The RRs were estimated for the following production types: indoor-layers (baseline); outdoor layers; broiler breeders; pullets; broilers; turkeys; duck meat and duck breeders. Dutch turkey and duck meat production types are all indoors.

We took British production types from the poultry demographic survey and used the most related definition described by Gonzales *et al*.^17^ to assign the RR of introduction of infection. We therefore had the following production types *j* (Dutch equivalent in brackets): indoor chicken; outdoor chicken (outdoor-layers); indoor turkey (turkey); outdoor turkey (outdoor-layers); indoor duck and goose (duck meat); outdoor duck and goose (duck breeders). There are no outdoor turkey holdings reported in the Netherlands, therefore we took the most applicable RR estimate for the outdoor turkey estimate, which was outdoor chicken. We assumed all partridge and pheasant holdings have at least some element of outdoors production, and we assumed that their susceptibility was most akin to outdoor chickens. The RRs are given in Table 1.

**Table 1:** Summary of the flock exposure parameter estimates for each production type *j*; modified from^17,18^. Risk Ratio is relative to P(indoor chicken), that is RR(indoor chicken) = 1.

Production type	Risk ratio, RR	*β*(*j*)
Indoor chicken	—	$6.17\times {10}^{-5}$
Outdoor chicken	11.1	$6.85\times {10}^{-4}$
Indoor turkey	7.7	$4.75\times {10}^{-4}$
Outdoor turkey	11.1	$6.85\times {10}^{-4}$
Indoor duck	12.8	$7.90\times {10}^{-4}$
Outdoor duck	24.5	$2.90\times {10}^{-3}$
Indoor goose	12.8	$7.90\times {10}^{-4}$
Outdoor goose	24.5	$2.90\times {10}^{-3}$
Outdoor partridge	11.1	$6.85\times {10}^{-4}$
Outdoor pheasant	11.1	$6.85\times {10}^{-4}$

Gonzales estimated the probability of introduction per month, which is related to the transmission parameter *β*(*j*) - the parameter used in the risk equation (see Eq. (3)) - by

4 $$P(j)=1-{e}^{-\beta (j)t},$$

where *t* is the number of months at risk (in the Dutch study 4 years = 48 months).

The relative risks between production types from the Dutch study (relative increase to P(indoor chicken) are given in Table 1). The absolute values of *β*(*j*) are anchored according to available UK data. There were 8/115 (7.0%) flocks found to be seropositive in the four years of poultry surveillance in the UK up to the end of December 2014, all of which were isolated from duck farms^19^. We used this information to anchor P(outdoor duck) at 0.07. The time period *j* of the model is arbitrary as we are primarily interested in relative not absolute risk, but we chose two months as a reasonable time period that would separate one incursion event from another. Rearranging Eq. (4), we therefore estimated the transmission parameter from wild birds to outdoor duck flocks, *β*(*outdoorduck*), to be 2.90 × 10^−3^. This compares reasonably well (in terms of order of magnitude) with the most comparable estimate from the Dutch study, duck breeders (which are mostly outside) of 8.6 × 10^−3^. We re-scaled the other estimates of *β*(*j*) using the baseline risk estimate of 2.90 × 10^−3^ for outdoor duck production and the RRs. The absolute values of *β*(*j*) used within the risk model are given in Table 1.

#### AI prevalence in wild birds

The UK does not currently conduct routine surveillance of healthy wild birds for AI. However a crude estimate of the prevalence of AI infection in wild birds can be made from EU wild bird surveillance data^16^. Of 9,302 healthy wild birds sampled across the EU in 2013 (the vast majority in Spain, Belgium and Germany), 12 were H5 positive, 29 were ‘Other LPAI’ positive and there were 101 ‘other positives’. Therefore, in the absence of more appropriate and detailed data, we set the prevalence of all AIs in British wild birds at 0.0152 (1.52%).

#### Model implementation and validation

We undertook a simple validation by observing the spatial distribution of AI incidents (both AI outbreaks and serological positives from the active poultry sero-surveillance over the same time period of 2011–2014, n = 8), and comparing it to the incursion risk map to see whether the spatial distribution of influenza A virus detections of AI infection correspond to areas of higher relative incursion risk. We chose to focus on outdoor ducks, as most of the UK seropositive detections, and all outbreaks, occurred on duck farms, and the highest risk group identified by the Dutch study were outdoor duck (breeder) farms^17^. This therefore provided as many positive samples as possible to map without confounding the spatial risk by inclusion of other poultry types.

The model was developed in R version 3.0.0, with packages ‘raster’, ‘rgdal’ and ‘maptools’.

## Results

### Wild bird abundance

We used a set of ‘ecological niche’ criteria to define 109 species for inclusion in the abundance calculations. The resulting abundance grid map for the species considered is shown in Fig. 2.

### Risk map

**Figure 3:**
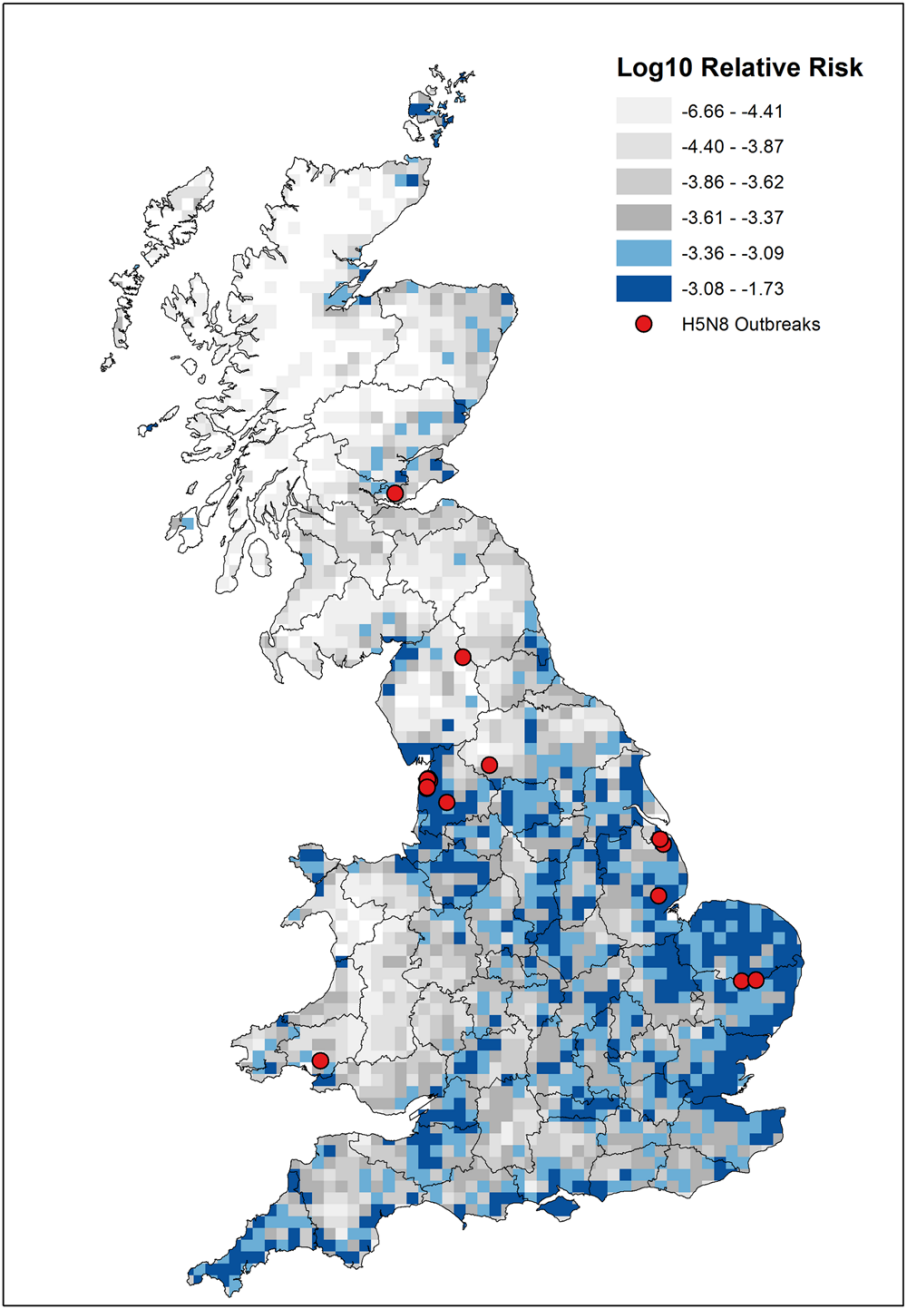
Relative risk map for the introduction of AI into poultry flocks from wild birds. The incursion risk is on a log scale. The visualisation scale is based on six equally distributed quantiles. For surveillance purposes poultry holdings are randomly sampled from the top two highest ranked quantiles (which represent 81% of total incursion risk). Red spot represents AI (specifically H5N8) outbreaks in period 2014–2017. Map was created from the results of our model in ArcGIS Desktop 10.2 (ESRI, Redlands, CA).

The updated risk map for the introduction of AI into GB poultry flocks via wild birds is presented in Fig. 3. We use six quantiles to visualise the risk across GB (similar to the previous 2006 risk map). The relative incursion risk is highly clustered, with most of the total risk of AI incursion in GB concentrated in specific areas of the country. The highest risk squares are of the order of 100,000 times the risk of the lowest non-zero risk cells. The darkest blue cells represent the top quantile (around 16 to 17% of all cells); these cells contain approximately 62% of total GB risk (where total GB risk is defined as the sum of risk over all grid squares). The top two quantiles (relative risk > log10–3.36) contains almost 81% of total GB risk.

Figure 3 also shows all AI outbreaks (all LPAI H5N8) that occurred during the timeframe of the analysis (2014–2017). While the number of incidents is too small to make a statistically significant assessment of spatial correlation, a calculation of a simple risk ratio (dividing average risk in cells with outbreaks by average risk in cells without any outbreaks) suggests that the average incursion risk is twice as high where outbreaks have occurred (RR = 2.07). While not conclusive, it does suggest that the model has some predictive power that can be used as a a guide for surveillance activities.

## Discussion

Pathogens that move across the interface between different populations, such as wildlife and livestock or animals and humans, pose a particular challenge to the development of effective and efficient surveillance and control measures. Avian Influenza viruses can move between wild birds, poultry and humans on a global scale with potentially devastating impacts. In this study, we developed a spatial targeting of surveillance for AI in wild birds and poultry in GB. The updated risk map presented in Fig. 3 represents an improved quantitative evaluation of the risk of introduction of AI into poultry flocks from wild birds over the previous study in 2007^8^. We quantified risk between grid cells by modifying the classical epidemiological risk equation, providing a strong quantitative foundation. We use contemporary, more robust and representative wild bird abundance estimates, as well as up to-date poultry holding information. Finally, the risk map is applicable to influenza A viruses in general, not specifically Eurasian-linage H5N1 HPAI. There have been several AI outbreaks on British poultry farms in the last few years, all due to HPAI H5N8, reinforcing the need for a surveillance tool that has breadth of virus strain coverage.

The results of the risk map suggest that the majority of incursion risk is clustered within certain areas of GB, including all of East Anglia (Suffolk, Norfolk and Cambridgeshire), Essex and Cornwall, as well as areas of Lancashire near the coast. Another cluster occurs in a strip of land straddling Derbyshire and Warwickshire. This clustering of high risk cells concentrates total GB risk in a reasonably small land area; the top 33% of cells by relative risk contribute over 80% of total GB risk. This suggests that targeted risk-based sampling in a relatively small geographical area could cover much of the risk presented by wild birds in GB. Many of the recent AI outbreaks in GB have been in these higher-risk areas, lending further credence to the value of targeted sampling in higher risk areas. Previous seropositive results from the surveillance of domestic ducks housed outdoors suggest that we have captured aspects that reflect higher exposure risk (RR = 2.07) and has some power to differentiate risk. We therefore believe our quantitative spatial method is robust enough to support the development of risk-based surveillance, assuming that wild birds are the primary or a major risk factor for the introduction of AI into British poultry flocks. Indeed, the primary suspected source of introduction for a number of recent AI outbreaks in GB, from November 2014 to January 2016, was wild birds^20–22^.

Of course, with any model there are uncertainties and assumptions that are required to be able to use and analyse available data. A key limitation of this type of risk map is the lack of detail around the association of wild bird species with poultry farms. We have excluded species restricted to coastal areas, but there are many other considerations for particular species that would mean contact between wild birds and poultry is more or less likely than that suggested by the fact that the species distribution overlaps poultry farming land. For example, Whooper Swans (Cygnus cygnus) migrate to and from Iceland and winter mostly in large wetlands and arable fields; there is limited potential for these birds to directly interact with poultry. We need to further research the interaction on or near poultry farms and the role of so-called ‘bridge species’ to better understand the interaction and hence transmission of AI between wild birds and poultry^23^. It is also apparent that different AI virus subtypes and pathotypes can show variation in the epidemiology in wild birds. For example, variation in species susceptibility and spatiotemporal infection dynamics will increase heterogeneity in the spatial risk^19^. However current knowledge and available data are not yet sufficient to fully incorporate this variation into risk models.

The results of this model therefore provide an updated and improved basis for risk-based surveillance. The model is actively being used by the British AI sero-surveillance programme in poultry. Samples are randomly taken from poultry holdings that reside in grid cells from the risk map’s top two quantiles (see Fig. 3). Additional risk factor data are then used to inform how many farms should be sampled within these areas (risk factors are whether a farm is in proximity to a water body, a farm is a mixed species farm with waterfowl present or is a free-range farm). The updated risk map described in this paper allows a much higher-resolution geographical direction of sampling and resources than previously, whilst still maintaining good coverage of the spatial risk in GB.

This improved method also provides a generic template for national surveillance programmes that may serve as a model for other pathogens at the wildlife-livestock interface such as classical swine fever, West Nile virus, Salmonella and rabies virus.

## Supplementary information

Supplementary information

## Data Availability

All freely available data are included in this manuscript. Gridded wild bird assemblage data are proprietary BTO data, although summary information is available in Musgrove *et al*.^14^. Gridded poultry holding data are not publicly available due to the UK Data Protection Act, but summary information at a county level is available from APHA (further information and contact details are available in APHA^13^).
