# Supplementary material for: Quantifying the spatial risk of Avian Influenza introduction into British poultry by wild birds
Source: Sci Rep. 2019 Dec 27;9:19973. doi: 10.1038/s41598-019-56165-9 (PMC6934731; doi:10.1038/s41598-019-56165-9)
Supplement: Supplementary file 1 — Supplementary information [file 41598_2019_56165_MOESM1_ESM.pdf]

Supplementary information for "Quantifying the spatial risk of Avian Influenza introduction into British poultry by wild birds"

By Andrew Hill, Simon Gillings, Alexander Berriman, Adam Brouwer, Andrew Breed, Lucy Snow, Adam Ashton, Charles Byrne and Richard M. Irvine.

Table A1: Assessment against criteria for 109 wild bird species. For population sizes, NP = Not Present (e.g. not present as a breeding species) and NE = population not estimated (e.g. in the case of resident breeding raptors for which there are no published wintering population estimates).

**Common name**	**Scientific name**	**UK Population size (individuals)**		**Criteria**					**Species selected**
		**Breeding**	**Winter**	**>1000 individuals waterbird**	**>100 individuals predator or scavenger**	**Terrestrial & freshwater habitats**	**Significant scavenger**	**Preys upon Anser. and Char.**	
Mute Swan	Cygnus olor	12,600	79,000	Y		Y			Y
Bewick's Swan	Cygnus columbianus	NP	7,000	Y		Y			Y
Whooper Swan	Cygnus cygnus	23	15,000	Y		Y			Y
Bean Goose	Anser fabalis	NP	730			Y			
Pink-footed Goose	Anser brachyrhynchus	2	360,000	Y		Y			Y
White-fronted Goose	Anser albifrons	NP	15,400	Y		Y			Y
Greylag Goose	Anser anser	92,000	228,000	Y		Y			Y
Snow Goose	Anser caerulescens	8	180			Y			
Canada Goose	Branta canadensis	124,000	190,000	Y		Y			Y
Barnacle Goose	Branta leucopsis	2,000	94,000	Y		Y			Y
Brent Goose	Branta bernicla	NP	121,400	Y		Y			Y
Egyptian Goose	Alopochen aegyptiaca	2,200	3,400	Y		Y			Y
Shelduck	Tadorna tadorna	30,000	66,000	Y		Y			Y
Mandarin Duck	Aix galericulata	4,600	7,000	Y		Y			Y
Wigeon	Anas penelope	800	450,000	Y		Y			Y
American Wigeon	Anas americana	NP	8			Y			
Gadwall	Anas strepera	2,420	25,000	Y		Y			Y
Teal	Anas crecca	4,400	220,000	Y		Y			Y
Green-winged Teal	Anas carolinensis	NP	25			Y			
Mallard	Anas platyrhynchos	213,000	710,000	Y		Y			Y
Pintail	Anas acuta	42	29,000	Y		Y			Y

Common name	Scientific name	UK Population size (individuals)		Criteria					Species selected
		**Breeding**	**Winter**	**>1000 individuals waterbird**	**>100 individuals predator or scavenger**	**Terrestrial & freshwater habitats**	**Significant scavenger**	**Preys upon Anser. and Char.**	
Garganey	Anas querquedula	107	NP			Y			
Shoveler	Anas clypeata	1,330	18,000	Y		Y			Y
Red-crested Pochard	Netta rufina	41	320			Y			
Pochard	Aythya ferina	980	48,000	Y		Y			Y
Ring-necked Duck	Aythya collaris	NP	18			Y			
Ferruginous Duck	Aythya nyroca	NP	10			Y			
Tufted Duck	Aythya fuligula	35,000	120,000	Y		Y			Y
Scaup	Aythya marila	NP	12,000	Y		Marine			
Eider	Somateria mollissima	55,000	58,000	Y		Marine			
Long-tailed Duck	Clangula hyemalis	NP	11,000	Y		Marine			
Common Scoter	Melanitta nigra	104	100,000	Y		Marine			
Surf Scoter	Melanitta perspicillata	NP	13			Marine			
Velvet Scoter	Melanitta fusca	NP	2,500	Y		Marine			
Goldeneye	Bucephala clangula	400	27,000	Y		Y			Y
Smew	Mergellus albellus	NP	180			Y			
Red-breasted Merganser	Mergus serrator	4,800	9,000	Y		Marine			
Goosander	Mergus merganser	6,900	12,000	Y		Y			Y
Ruddy Duck	Oxyura jamaicensis	60				Y			
Honey-buzzard	Pernis apivorus	102	NP		Y	Y			
Red Kite	Milvus milvus	3,200	NE		Y	Y	Y		Y
White-tailed Eagle	Haliaeetus albicilla	81	NE			Y	Y	Y	Y1*
Marsh Harrier	Circus aeruginosus	700	NE		Y	Y		Y	Y
Hen Harrier	Circus cyaneus	1,290	NE		Y	Y		Y	Y

Common name	Scientific name	UK Population size (individuals)		Criteria					Species selected
		Breeding	Winter	>1000 individuals waterbird	>100 individuals predator or scavenger	Terrestrial & freshwater habitats	Significant scavenger	Preys upon Anser. and Char.	
Montagu's Harrier	Circus pygargus	28	NP			Y			
Goshawk	Accipiter gentilis	710	NE		Y	Y		Y	Y
Sparrowhawk	Accipiter nisus	66,000	NE		Y	Y		Y	Y
Buzzard	Buteo buteo	141,000	NE		Y	Y	Y		Y
Rough-legged Buzzard	Buteo lagopus	NP	32			Y		Y	
Golden Eagle	Aquila chrysaetos	880	NE		Y	Y		Y	Y
Osprey	Pandion haliaetus	450	NP		Y	Y			
Kestrel	Falco tinnunculus	94,000	NE		Y	Y			
Merlin	Falco columbarius	2,400	NE		Y	Y		Y	Y
Hobby	Falco subbuteo	5,600	NP		Y	Y			
Peregrine	Falco peregrinus	3,000	NE		Y	Y		Y	Y
Oystercatcher	Haematopus ostralegus	220,000	340,000	Y		Y			Y
Black-winged Stilt	Himantopus himantopus	1	NP			Y			
Avocet	Recurvirostra avosetta	3,000	7,500	Y		Y			Y
Stone-curlew	Burhinus oedicnemus	700	NP			Y			
Little Ringed Plover	Charadrius dubius	2,500	NP	Y		Y			Y
Ringed Plover	Charadrius hiaticula	10,900	34,000	Y		Y			Y
Dotterel	Charadrius morinellus	1,260	NP	Y		Montane			
Golden Plover	Pluvialis apricaria	97,800	420,000	Y		Y			Y
Grey Plover	Pluvialis squatarola	NP	43,000	Y		Intertidal			
Lapwing	Vanellus vanellus	280,000	650,000	Y		Y			Y
Knot	Calidris canutus	NP	330,000	Y		Intertidal			
Sanderling	Calidris alba	NP	17,000	Y		Coastal			
Little Stint	Calidris minuta	NP	14			Y			

Common name	Scientific name	UK Population size (individuals)		Criteria					Species selected
		Breeding	Winter	>1000 individuals waterbird	>100 individuals predator or scavenger	Terrestrial & freshwater habitats	Significant scavenger	Preys upon Anser. and Char.	
Purple Sandpiper	Calidris maritima	2	13,000	Y		Coastal			
Dunlin	Calidris alpina	19,200	360,000	Y		Y			Y
Ruff	Calidris pugnax	11	820			Y			
**Common name**	**Scientific name**	**UK Population size (individuals)**	**Criteria**	**Species selected**					
Jack Snipe	Lymnocryptes minimus	NP	110,000	Y		Y			Y
Snipe	Gallinago gallinago	162,000	1,100,000	Y		Y			Y
Woodcock	Scolopax rusticola	161,000	1,400,000	Y		Y			Y
Black-tailed Godwit	Limosa limosa	127	44,000	Y		Y			Y
Bar-tailed Godwit	Limosa lapponica	NP	41,000	Y		Intertidal			
Whimbrel	Numenius phaeopus	900	30			Montane			Y2*
Curlew	Numenius arquata	138,000	150,000	Y		Y			Y
Common Sandpiper	Actitis hypoleucos	30,000	73	Y		Y			Y
Green Sandpiper	Tringa ochropus	4	910			Y			
Spotted Redshank	Tringa erythropus	NP	98			Y			
Greenshank	Tringa nebularia	2,200	770	Y		Y			Y
Wood Sandpiper	Tringa glareola	38	NP			Y			
Redshank	Tringa totanus	50,000	130,000	Y		Y			Y
Turnstone	Arenaria interpres	NP	51,000	Y		Coastal			
Red-necked Phalarope	Phalaropus lobatus	44	NP			Y			
Arctic Skua	Stercorarius parasiticus	4,200	NE	Y	Y	Marine			
Great Skua	Stercorarius skua	19,200	NE	Y	Y	Marine		Y	
Kittiwake	Rissa tridactyla	760,000	NE	Y	Y	Marine			
Black-headed Gull	Chroicocephalus ridibundus	280,000	2,200,000	Y	Y	Y	Y		Y

Common name	Scientific name	UK Population size (individuals)		Criteria					Species selected
		Breeding	Winter	>1000 individuals waterbird	>100 individuals predator or scavenger	Terrestrial & freshwater habitats	Significant scavenger	Preys upon Anser. and Char.	
Little Gull	Hydrocoloeus minutus	1	NE			Marine			
Mediterranean Gull	Larus melanocephalus	1,230	1,800	Y	Y	Y	Y		Y
Common Gull	Larus canus	98,000	710,000	Y	Y	Y	Y		Y
Ring-billed Gull	Larus delawarensis	NP	22			Y	Y		
Lesser Black-backed Gull	Larus fuscus	220,000	130,000	Y	Y	Y	Y		Y
Herring Gull	Larus argentatus	280,000	740,000	Y	Y	Y	Y	Y	Y
Yellow-legged Gull	Larus michahellis	2	1,100	Y	Y	Y	Y		Y
Caspian Gull	Larus cachinnans	NP	90			Y	Y		
Iceland Gull	Larus glaucoides	NP	240		Y	Y	Y		Y
Glaucous Gull	Larus hyperboreus	NP	170		Y	Y	Y		Y
Great Black-backed Gull	Larus marinus	34,000	77,000	Y	Y	Y	Y	Y	Y
Chough	Pyrrhocorax pyrrhocorax	935	NE		Y	Y			
Magpie	Pica pica	1,180,000	NE		Y	Y	Y		Y
Jay	Garrulus glandarious	358,200	NE		Y	Y			
Jackdaw	Corvus monedula	2,700,000	NE		Y	Y			
Rook	Corvus frugilegus	2,200,000	NE		Y	Y			
Carrion Crow	Corvus corone	2,000,000	NE		Y	Y	Y		Y
Hooded Crow	Corvus cornix	400,000	NE		Y	Y	Y		Y
Raven	Corvus corax	15,000	NE		Y	Y	Y		Y

*Included in species list due to likely agreement with criteria if young included in numbers.
